# Rapid quantitative analysis of double-stranded plasmid DNA with capillary gel electrophoresis for applications in quality control and radiation research

**DOI:** 10.1038/s41598-025-85132-w

**Published:** 2025-01-07

**Authors:** Marc Benjamin Hahn

**Affiliations:** 1 Institut für Chemie, Universität Potsdam, 14476 Potsdam, Germany; 2 Bundesanstalt für Materialforschung und -prüfung, 12205 Berlin, Germany

**Keywords:** Biological physics, Molecular biophysics, Biophysical methods, Isolation, separation and purification

## Abstract

The quantification of different structures, isoforms and types of damage in plasmid DNA is of importance for applications in radiation research, DNA based bio-dosimetry, and pharmaceutical applications such as vaccine development. The standard method for quantitative analysis of plasmid DNA damage such as single-strand breaks (SSB), double-strand breaks (DSB) or various types of base-damage is Agarose gel electrophoresis (AGE). Despite being well established, AGE has various drawbacks in terms of time consuming handling and analysis procedures. A more modern, faster, cheaper and more reliable method is capillary gel electrophoresis (CGE). However, to establish this method in biotechnology, radiation-research and related fields, certain criteria in terms of accuracy, repeatability and linearity have to be tested and protocols have to be established. This study performs the relevant tests with a common model plasmid (pUC19, double-stranded DNA with 2686 basepairs) to establish a CGE based methodology for quantitative analysis with readily available commercial CGE systems. The advantages and limitations of the methods are evaluated and discussed, and the range of applicability is presented. As a further example, the kinetics of enzyme digestion of plasmid DNA by capillary gel electrophoresis was studied. The results of the study show for a model system consisting out of pUC19, the suitability of CGE for the quantification of different types of DNA damage and the related isoforms, such as supercoiled, open-circular and linear plasmid DNA.

## Introduction

Structural integrity of covalently-closed circular (CCC)/supercoiled (SC) plasmid DNA is of importance for a wide range of fields, spanning from pharmacy, over biotechnology, to nanotechnology^1^. For example, double-stranded plasmid DNA plays an important role in pharmaceutical research, for gene therapy, vaccine development^2–7^, and the study of effects of radio sensitizers for cancer treatment^8–10^. In novel approaches for biodosimetry^11–13^ and radiation biophysics^14–17^, plasmid DNA is often evaluated in terms of DNA strand-break induction. Hereby, all these applications have in common, that they require an intact DNA sugar-phosphate backbone, which provides the structural integrity of the DNA molecule^18^. Damage to this DNA backbone can lead to DNA strand-breaks, and in the worst case, to fragmentation of the molecule.

To control, test and quantify the various structures of plasmid DNA, reliable, fast and cost efficient methods, which are suitable for routine operations, are required. Currently the standard method to quantify the different structural forms of plasmid DNA in a sample is Agarose-gel electrophoresis (AGE). It is well established and available in many biochemical laboratories. However, there are various drawbacks to this method. AGE involves many manual preparation steps from a lab technician, and thus the outcome and uncertainties of a quantitative analysis often depends on the skills of the technician involved, and therefore is strongly dependent on a “human factor”. Furthermore, it is time consuming, has a low degree of automatization and often requires handling of DNA dyes which are partly suspected to be carcinogenic.

However, within the last years, capillary gel-electrophoresis (CGE) has been developed as an alternative^3,19,20^ and become more widely available^21^. It is a well established technique to perform analyses of highly fragmented DNA^33^. In contrast, the possibility to perform quality control for only slightly alterations in plasmid DNA samples, to test their structural integrity and quantify different plasmid isoforms was investigated inititally by Schmidt et al.^19^ Furthermore, Mitchenall et al.^21^ analyzed different topological forms of plasmid DNA, to investigate the reactions of DNA topoisomerases to plasmid DNA in high-resolution by CGE, which would be very challenging with conventional AGE.

Despite their higher-initial costs, they provide lower cost per sample, less manual steps in performing an analysis, no manual handling of carcinogenic substances, and promise higher reproducibility for quantitative analysis, as will be tested below.

To establish the related methodologies for quantitative analysis of plasmid DNA in the various fields, a calibration methodology and respective reference data have to be provided. Herein we provide the description of a testing methodology and analysis to establish high-resolution capillary gel-electrophoresis to determine the amount of different isoforms of plasmid DNA in a model system consisting of double-stranded pUC19 with 2686 basepairs (bp) with focus on practical applications in radiation research and quality control, where in both cases a high degree of structurally intact plasmids must be present. This system consisting of plasmid pUC19 has been selected since it is well studied^3,11,21^, serves as a model system in radiation research^11,16^ and is currently being evaluated in for applications as biodosimeters^1^.

**Fig. 1:**
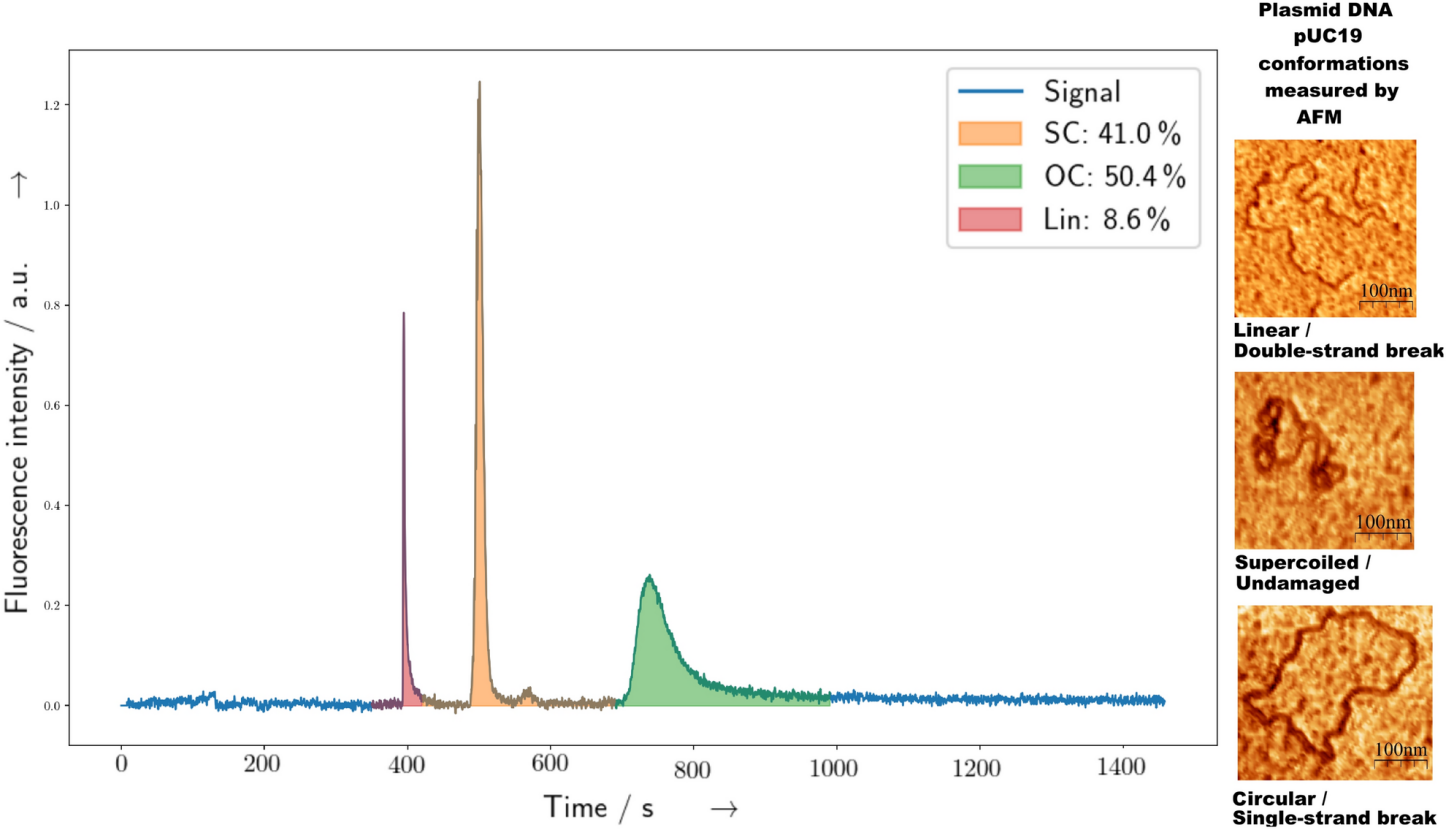
Plasmid conformations and their position in CGE densiograms. Left: Densiogram of plasmid pUC19 with 2686 bp and three clearly visible isoforms, shown from left to right as linear (Lin: red, around 400 s), supercoiled/covalently-closed circular (SC: yellow, around 500 s) and open-circular (OC: green, around 750 s) conformation. Right: pUC19 isoforms measured by atomic-force microscopy (AFM). The AFM images were taken and modified with permission from^1^.

Furthermore it is similar to other common plasmids such as *pUC18*, *pGEM* or *pRB* in the size range of around 2000–5000 bp. These undamaged CCC-DNA systems are ideal model systems to study DNA degradation because their covalently closed form is topologically constrained, resulting in a compaction of the molecule, leading to a fast migration during chromatography experiments. This compact CCC/supercoiled form of the plasmid pUC19 is shown in Fig. 1 right center as an example.

When a single strand break (SSB) occurs in the sugar-phosphate backbone of such a CCC-DNA, it is less constrained, and therefore relaxes in the case of the double-stranded plasmid DNA to the open-circular (OC) form as shown in Fig. 1 right bottom. After induction of a double-strand break (DSB), the plasmid dsDNA undergoes an additional change, into the linear conformation as shown in Fig. 1 right top. In contrast to that, in single-stranded phage DNA in its covalently closed form, only a SSB can occur, which leads to formation of a linear ssDNA molecule. All these DNA forms can be separated and quantified by chromatographic methods such as AGE and CGE. During such a separation, the different damaged and undamaged species corresponds to different signals in the chromatogram as shown in Fig. 1. However, it cannot be assumed *a priori*, that the same amount of species obeys the same linear relation between quantity and the detected signal during a measurement. Therefore, these relation between measured species, detector response and output signal have to be determined by a calibration procedure for the respective combination of samples, devices and detection method. To allow CGE to be used as an replacement for AGE for quantitative measurements, the equivalence of the results, the concentration dependence, the reproducibility, and behaviour of different isoforms have to be studied and a reliable protocol has to be established. This will be performed in the following.

## Results and discussion

The structural stability and respective damage of double-stranded (dsDNA) DNA is related to the integrity of the DNA sugar-phosphate backbone. In CCC/SC plasmid DNA the and individual breakage of the sugar-phosphate backbone is linked (single-strand break, SSB) to the conversion of the very compact and topological constrained form of the plasmid (Fig. 1 right, center) to the relaxed OC form (Fig. 1 right, bottom) plasmid DNA to the open-circular (OC) form. When two SSB occur on opposite strands of dsDNA within a short distance of only some base-pairs, a so called double-strand break (DSB) can lead to an additional structural change, leading to the linearization of the plasmid (Fig. 1 right, top). Compared to SSB or simple base-damages, this type of DNA damage is more difficult to repair and thus of higher biological relevance^16^.

To understand and quantify these damages is a common goal in radiation research and related fields^22^. Here, it is beneficial, that the topological isoform of the plasmid can be easily measured by chromatographic methods such as AGE or CGE, while it is directly linked to modifications of individual covalent bonds in the sugar-phosphate backbone of the plasmid DNA^1^.

Besides the structural stability of the DNA molecule, the integrity of the DNA bases and their sequence is all-important for their role in storing the genetic code. However, change in the DNA bases are not directly visible in chromatographic experiments. Nevertheless, they can be observed after treating the plasmids with enzymes (*e.g* T4, Nth, or FPG), which convert certain types of base-damage into DNA strand-breaks^15,23^. Therefore, the methods described herein is beneficial in studying various types of base damage and degradation, going beyond strand-breaks alone.

To evaluate the possibility to use CGE as a replacement for AGE to quantify damage in plasmid DNA, various types of analyses under different conditions for the same set of samples were performed simultaneously with both methods.

As a prerequisite the assignment of the measured signals to the most relevantly plasmid isoforms had to be performed. Therefore, pUC19 plasmid DNA with a high-initial degree of CCC/SC form (SC $\geq 90\,\%$ ) was obtained and tested by AGE. Different restriction enzymes were applied to convert to produce artificial samples with purely linear and open-circular (OC) form. These samples were analysed and quantified by both AGE and CGE. Exemplary CGE and AGE measurements for comparing SC and OC plasmid DNA are shown in Fig. 1.

**Fig. 2:**
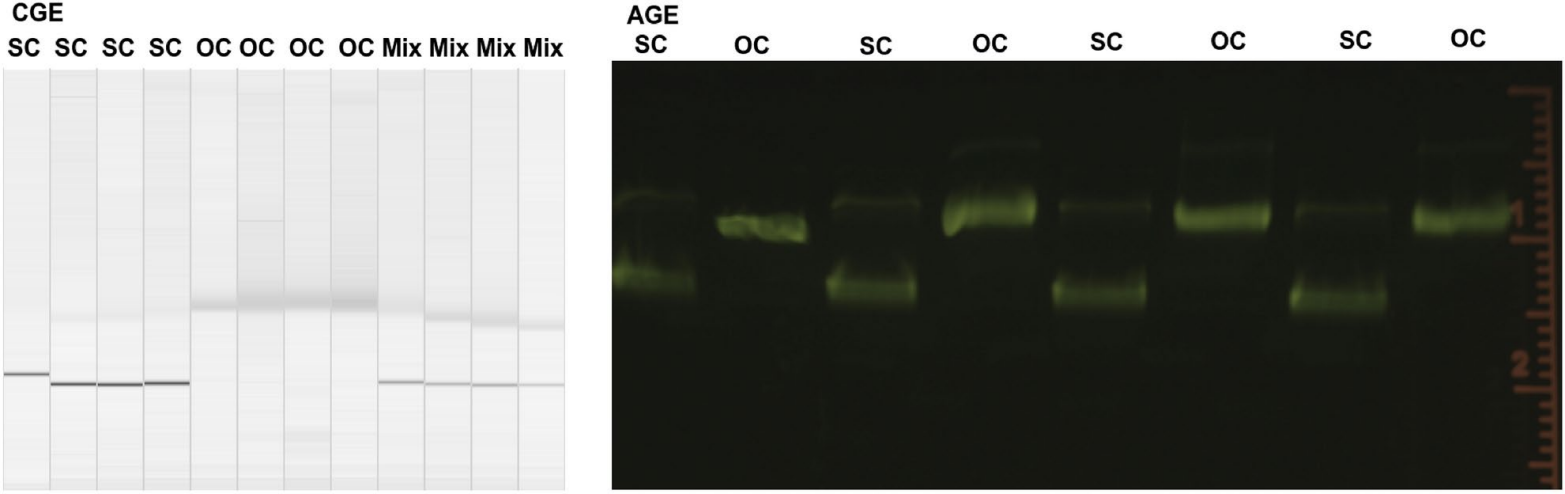
Comparision of AGE and CGE running behaviour with different forms of plasmid pUC19. On the left a Qiaxcel-CGE generated image from the CGE measurement at pUC19 is shown. This resembles similar properties as the traditional AGE images taken from an Agaorse gel as shown on the rightern side. In both samples the lanes of non-digested (SC) plasmid DNA in mostly supercoiled form and the digested (OC) open-circular form are displayed. In case of the CGE image, additional lanes (Mix) are present, containing 1:1 mixtures from SC and OC samples. It is apparent in the CGE measurements, that the bands assigned to SC forms have a higher peak intensity with much smaller width than the peaks related to the OC form. This is evident as well in the CGE densiogram displayed on the leftern side of Fig. 1. Please note that all lanes contain only plasmid DNA pUC19 which only differe in their structure being either only SC, OC or a mixture of SC and OC plasmid. These forms are visualized as well on the rightern side of Fig. 1.

**Fig. 3:**
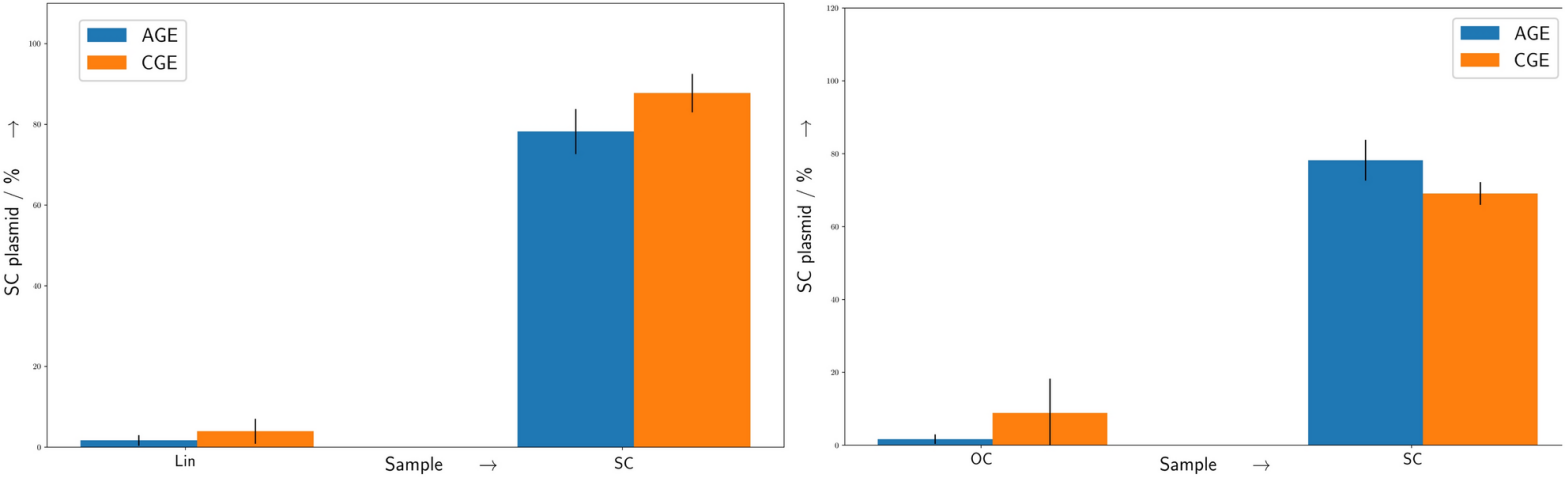
Quantification of OC and linear plasmid DNA in AGE and CGE. Comparison of AGE (blue) and CGE (orange) measurements for the quantification of linear (left) and open-circular (right) plasmid DNA produced by enzyme digests. Shown are the undigested control samples (SC) and enzyme digested samples as OC and Lin, respectively. Four technical replicates were each measured three times. Errorbars represent the standard deviation. For details see the text.

**Fig. 4:**
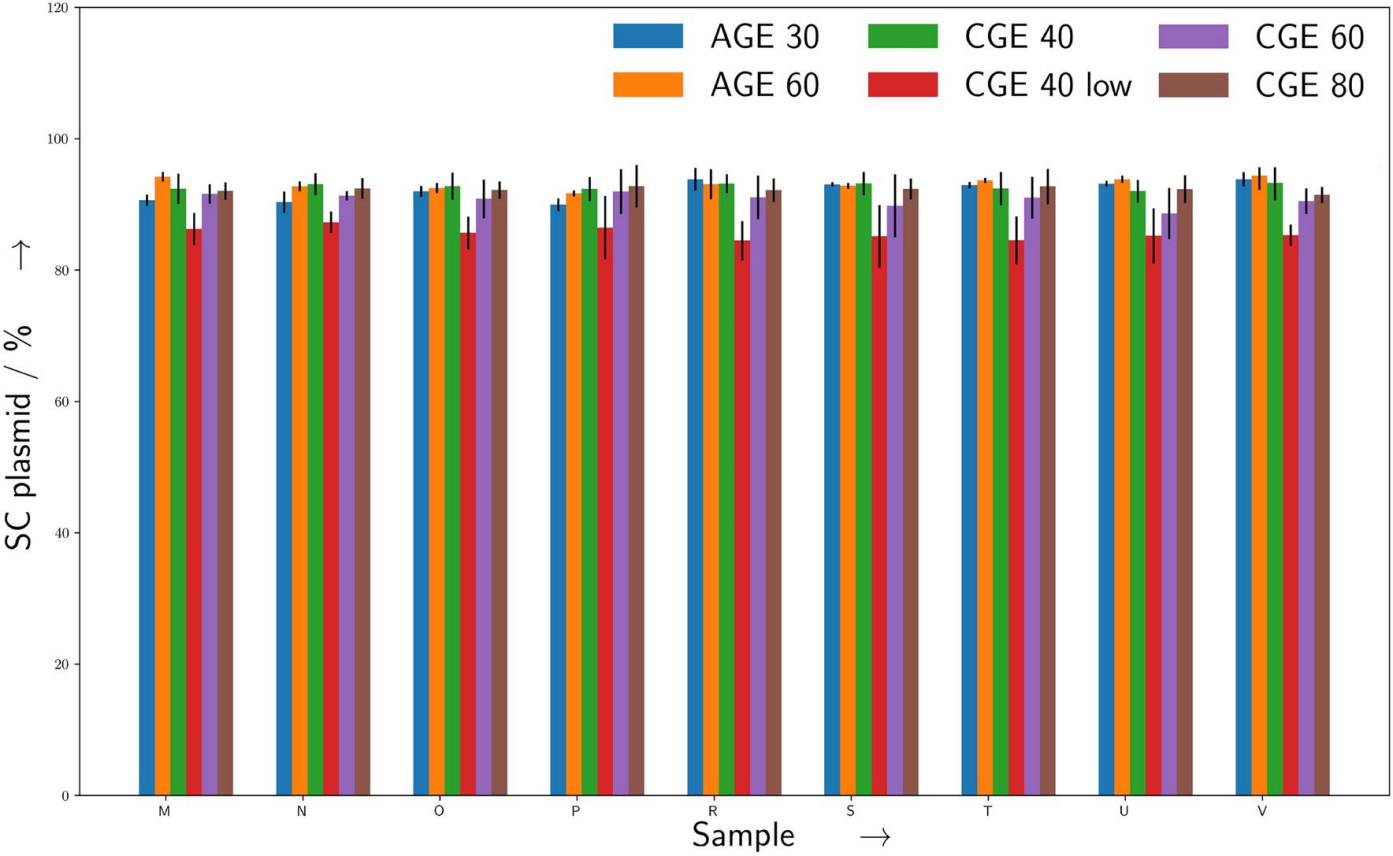
Comparision between CGE and AGE for different running parameters. Shown is the relative amount of undamaged (SC) plasmids within a sample. The data provides a comparison of AGE and CGE measurements with nine replicates, as shown along the x axis (M-V), of samples from the same plasmid batch. The AGE measurements (n = 6 lanes per replicate) were taken after 30 min (blue) and 60 min (orange). CGE measurements were taken for injection times of 40 s (green, red) 60 s (purple) and 80 s (brown) (n = 8 per replicate). Errorbars represent the standard deviation. For details see the text.

There, the leftern part of the figure shows the CGE measurements in a similar representation as common for AGE. Hereby the first four lanes contained only the untreated plasmid DNA, which show a unique, well defined single band corresponding (compare as well Fig. 1 yellow region around 500 s) to the compact unique SC form. The four central lanes contained a completely enzyme treated samples to produce a sample with purely OC conformation. The corresponding, slower migrating band is less intense with a broader width, as it is as well evident in the exemplary densiogram in Fig.1, shown as green marked region with a migration time of around 750 s. The latter four lanes (Mix) contain a 1:1 mixture of SC and OC DNA. The corresponding bands are both visible here with decreased intensity. As a control the same SC and OC samples were measured by AGE (rightern side) in alternating order as indicated above the gel image. Here, the well known SC and OC bands are visible as expected. The reproducibility in terms of peak positions within the same cartridge capillary, injection, and run parameters, on the same sample is given, while they can differ between the different capillaries as evident in Fig. 2. However, the intra-peak distances, and more importanlty the quantitative analysis results within repeated measurements of the same capillary and sample are in the range of single digit percentage values (compare Fig. 4).

Additional to the experiments comparing plasmids in SC and OC form, the same experiments were performed with linearized plasmid DNA. Here a very sharp peak, corresponding to the linear form of the plasmid is visible in the densiogram (Fig. 1 red region around 400 s). For both experiments, the respective untreated and digested samples were analyzed by AGE and CGE and the quantification results are shown in Fig. 3 Results from both methods show similar quantitative values. However, it is noteworthy here, that the SC degree of the control sample in both experiments (Fig. 3 Linearized, left and Circularized, right) is lower for the circularized samples. The most likely explanation for this behaviour is related to the increased exposure to higher temperatures during the procedure of the enzyme digest with *Nt.BspQI* compared to *HindIII* (see “Materials and methods” Section). These prolonged higher temperatures may convert more of the heat-labile sites^14^ in the plasmid DNA to strand-breaks, even for the blind samples, where no enzyme was present. As an additional test to compare the overall accuracy of the CGE measurements with standard AGE measurements, nine replicates of untreated plasmid DNA samples were analyzed simultaneously by both methods with various setting. For the AGE experiments standard conditions with two different runtimes were chosen^1^, while for the CGE measurement an optimized profile with an extended runtime for the Qiaxcel standard cartridge was obtained from the manufacturer. The exact parameters are described in the “Materials and methods” Section.

The comparison shown in Fig. 4 of the AGE measurements with 30 and 60 min runtime with CGE measurements with varying injection times of 40, 60 and 80 s that the quantification of the relative percentage of undamaged/SC/CCC-form of plasmid DNA pUC19 is in good agreement for all samples, with the exception of the *CGE 40 low, red* dataset corresponding to an CGE injection time of 40 s, which was measured at the same samples directly after the eight *CGE 60* and eight separate *CGE 80* measurements.

It turned out that after the measurement the approximately eight hours needed for measuring the complete *CGE 60* and *CGE 80* datasets, some microliter of the originally 10 μL containing samples were evaporated in the rather low-humidity lab environment, which substantially increased the signal-noise ratio (SNR) within the densiograms, and therefore decreased the quality of the data, as visible in the lowered accuracy of the *CGE 40 low, red* dataset. However a freshly prepared set of samples lead as well for the repeated measurements with 40 s injection time (*CGE 40, green*) to results which were in good agreement with the other measurements. We still decided to include the *CGE 40 low* dataset to highlight the importance of checking the correct filling of the sample tubes with at least 10 μL of liquid, the absence of bubbles, and proper centrifuging before the measurements.

**Fig. 5:**
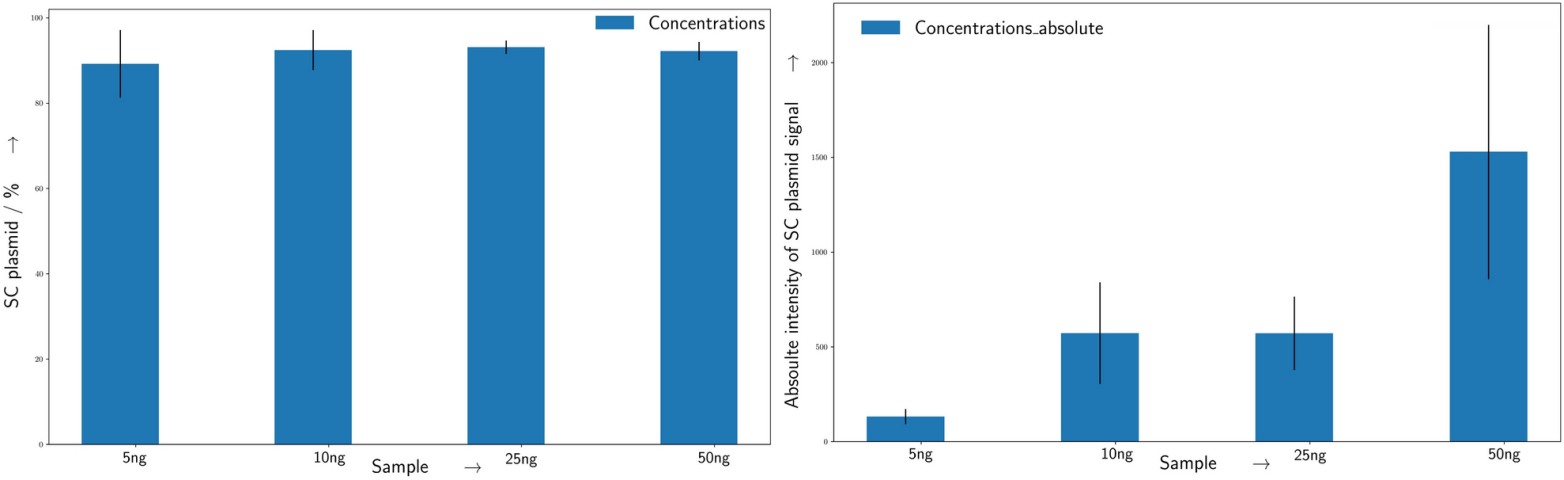
Comparision of relative and total signal intensity in CGE. Left: Shown are the quantification results of the relative amount of CCC/SC form of pUC19 for different DNA concentrations as shown on the x axis. Within the given range the results are concentration independent and in good agreement with each other. Right: The total signal intensity is shown in dependence of the DNA concentration. A correlation between concentration and integrated signal intensity is apparent, but the high scattering of the absolute intensity values does not permit a determination of the actual DNA concentration. Errorbars represent the standard deviation (n = 3).

The analysis method was tested for different DNA concentration between 5, 10, 25, and 50 ng/μL. Higher DNA concentrations with high degree of SC or linear conditions can lead to saturation of the detector signal at the respective band, making a quantification impossible. In this case a dilution of the samples is needed. Thus, for the reduced concentrations, the results for the relative amount of SC plasmids within a sample were independent of the DNA concentrations in the sample (Fig. 5 left.). Only the resulting uncertainty calculated from the standard deviation of the replicates increased with decreasing DNA concentration. Which is again attributed to the differences in SNR. With respect to the total signal intensity, a correlation can be observed between DNA concentration and overall signal intensity (Fig. 5 right). However the scattering of the values is far too huge as it can be used as a mean to determine and or even compare concentrations between the different samples.

Two questions, which are of special relevance for radiation research, are the clear separation between linear and and open-circular DNA and the linearity for determining accurate trends of the conversion between SC to OC form when the dose-damage relationship is studied^11,16^. The straight forward separation between OC and Linear conformation is often problematic in AGE measurements due to the often occurring partial overlap of the related bands in the gel. However, as clearly shown in Fig. 1, all three contributions are well separated in the CGE measurements. Compared to AGE, this property alone provides a huge advantage of CGE measurements, especially when quantitative analysis of samples with high OC and linear content has to be performed.

**Fig. 6:**
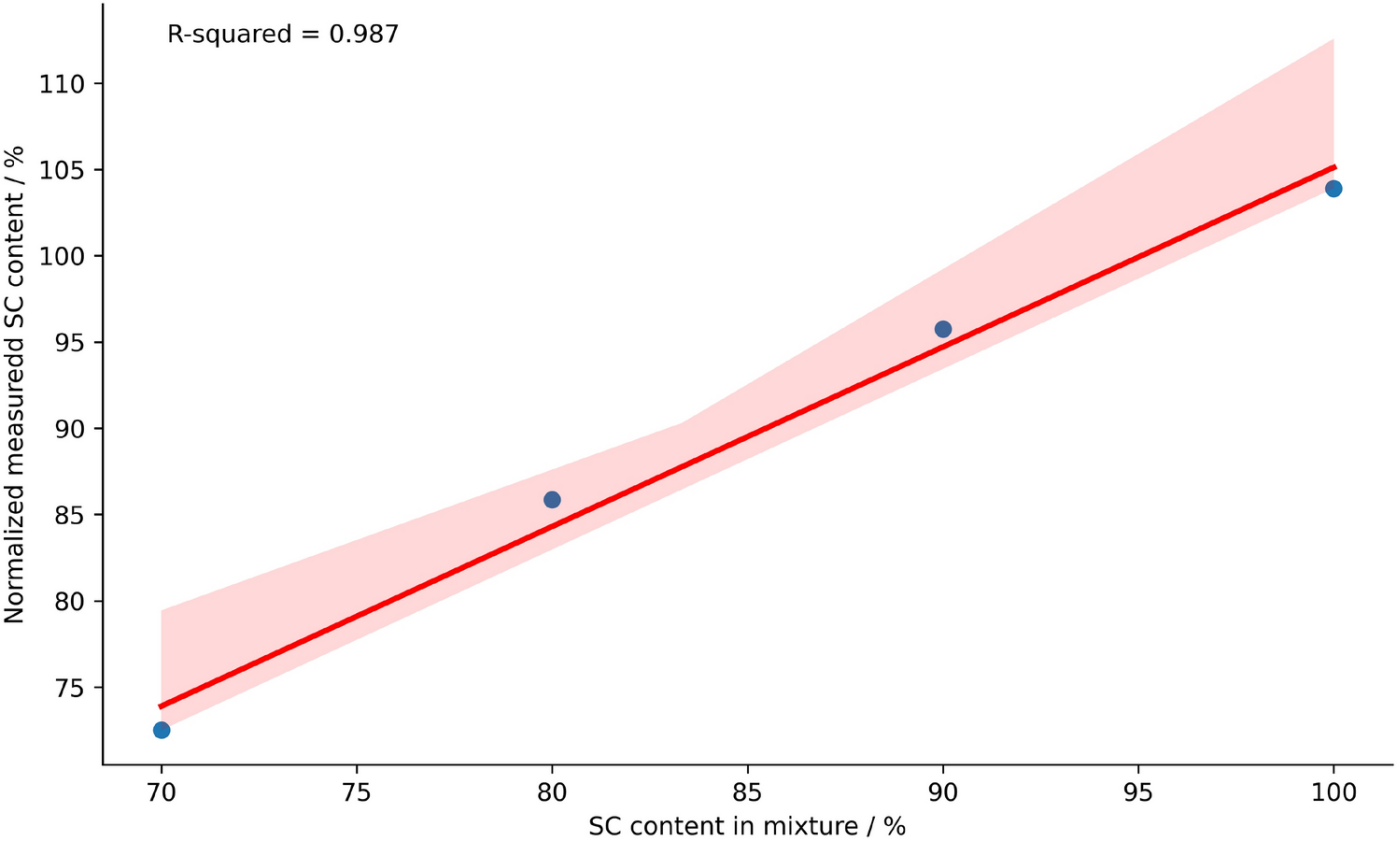
Linearity of the signal response in dependence of the amount of SC plasmid DNA. The relation between prepared SC content and determined SC content to test the linearity of the relation. The measured SC contents were normalized on the samples without added enzyme treated plasmids (100% SC content on the x axis). The red lines shows the results of the linear regression. The linear regression resulted in an $\hbox {R}^2$ = 0.987 showing a good agreement of the linear model with the data.

To perform reliable quantitative analysis of samples with low variations in dose and related damage induction, the linearity in the signal response during CGE measurements is of importance. Then, the necessity of repeatedly performing calibration procedures which represent an additional source of uncertainty can be avoided. To test this behaviour, various samples with different relative amount of untreated SC samples, and enzyme digested OC samples were mixed in different ratios and measured simultaneously. The measured and normalized SC content is displayed in Fig. 6 in dependence of the ratio of the SC:OC mixture as prepared. The data is shown together with a linear fit (red line) stemming from linear regression with a $\hbox {R}^2$ = 0.987. This indicates a linear increase of measured SC content with respect to the SC content in the mixture within the accuracy of the method.

**Fig. 7:**
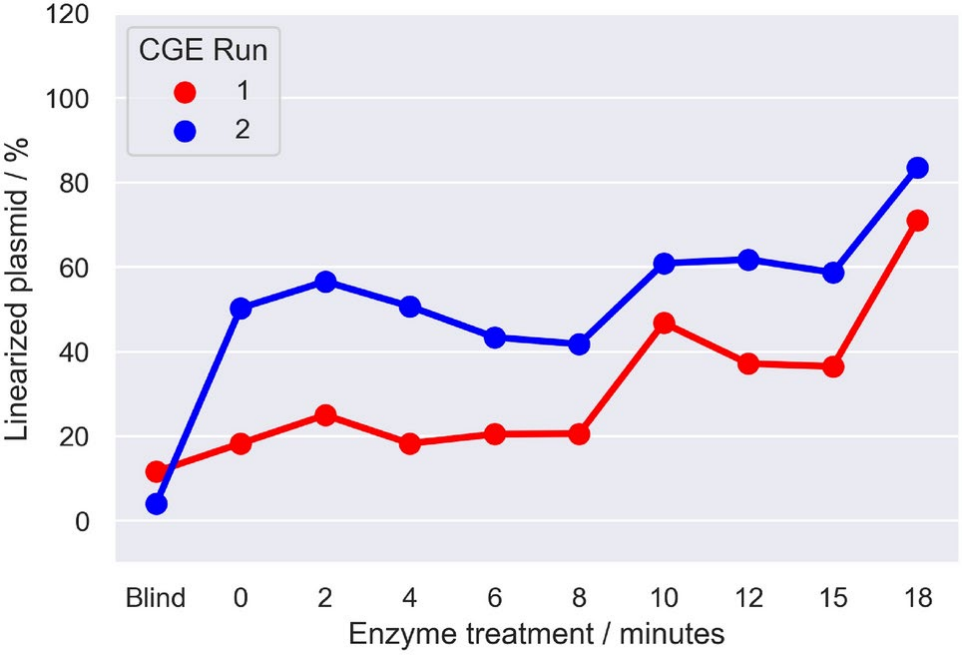
Time dependent HindIII digest of pUC19 monitored by CGE. All samples were kept for 18 min at 37 °C. The enzyme was added after varying timesteps to the samples, resulting in different enzyme treatment times as indicate on the x axis. Two CGE runs were performed on the same samples. The first run was directly performed after the incubation with HindIII (red), the second run (blue) 25 min later after the start of the first measurement . HindIII was not deactivated, thus additional action of the enzyme was observed between the two runs, as appearent in the higher amount of plasmid in the linear conformation in the blue curve and the difference between the Blind (no enzyme) and sample at t = 0 min, where the enzyme was added at the end of the incubation period.

To test whether CGE is capable to study the kinetics of DNA digesting enzymes over time SC pUC19 was incubated for different timespans with *HindIII* to convert the plasmid to its linear form. The samples were analyzed once directly after incubation and a second time aboyt 25 min later, after the first run. The results are shown in Fig. 7. It is clearly evident, that the degree of linearization of the plasmid increases with increasing incubation time at 37 °C as indicated along the x axis. Furthermore the difference between the first (red curve) and second (blue curve) show that within the 25 min between the measurements, where the samples were kept at room temperature (23 °C) further linearization happened but with decreased efficiency. This exemplary measurements show to feasiability to apply CGE for time dependent studies when time resolution in the range of minutes is needed. Other possible are the study of temperature or concentration dependent reactions, varying heating, enzyme or salt content instead of time. Especially the ability of repeateadly performing measurements at the same sample after a first run is of benefit here.

## Summary and outlook

In conclusion, it was shown that commercially available CGE systems can be used to easily separate and quantify the most relevant isoforms of standard plasmid DNA such as pUC19 in the range of around 2000-3000 bp. The efficient and clear separation of the three isoforms, related to undamaged plasmid DNA in its supercoiled form, plasmid DNA with a single-strand break in the open-circular form, and plasmids with a double-strand break in the linear form, represent a great advantage over classical Agarose-gel electrophoresis measurements. In addition, the linearity of the signal response, the fast experimental runs, repeatability and flexibility, show that CGE provides an efficient and reliable way of quantifying DNA isoforms in mixed samples, while at the same time decreasing the amount of manual, error prone sample handling steps in the laboratory. Furthermore, in contrast to AGE, no additional gel staining procedures with possibly carcinogenic DNA dyes is needed. Additionally, the data analysis of the CGE densiograms can be easily automatized, which stands in stark contrast to the manual analysis of gel images obtained by AGE on a variety of scanning systems. This provides an additional advantage when dealing with a huge set similar plasmid DNA, treated under varying conditions as they often occurr in radiation research when one deals with a wide parameter space.

Besides, of the approximately 10 μL needed during the CGE analysis, only less than one microliter is consumed by the CGE measurement itself, providing the possibility of performing additional independent analysis on the exact same aliquot of the sample. The only obvious drawback are the initial higher cost compared to classical AGE equipment, which are on the other hand counter balanced by a decreased working time to be spend in the laboratory for analysing the same amount of samples.

For the future, advancing the quantitative analysis of DNA with CGE beyond isolated plasmids is of interest for further fields. Following studies could involve similar DNA structures such as minicircle DNA in the range of 4 kbp. These would need new protocols and run profiles with extended duration due to their decreased electrophoretic mobility compared to the much shorter plasmid DNA studied here.

Additional to the already established CGE methods for studying the effects of topoisomerases on plasmids^21,24^, the the quantitative study of protein-DNA interaction as normally performed by electro-mobility shift-assay (EMSA) or fluorescence based methods^25^ would be of interest. Especially when the recent developments in the field of ultra-high dose rate ratiotherapy (FLASH) to treat cancer are taken into account^26,27^. To understand of radiation damage under the FLASH irradiation conditions the study of DNA, protein, lipids, and their interaction was indentified as highly relevant^27,28^. Here, especially the formation of DNA-protein crosslinks have to be quantified. Besides *in-situ* analysis of these effects, fast and reliable quantitative post irradiation analysis is needed^29–31^. For such analysis CGE could play a vital role in quantifying radiation induced covalent DNA-protein crosslinks, which are predominantly formed under low oxygen concentrations, as can be found in cancerous tissue with high radiation resistance^32^.

## Materials and methods

### Double-stranded plasmid DNA pUC19

Plasmid DNA pUC19 was obtained from Plasmidfactory (Bielefeld, Germany). There, the plasmids were cultivated in *E. coli* (K12), isolated and afterwards purified via multiple steps of chromatography. Resulting in a master batch with a total amount of 100 mg of pUC19 in 1 $\times$ PBS with a concentration of 0.48 mg/mL. The homogenization of the purified sample was achieved by gentle swirling. Afterwards 400 aliquots with 500 μL each were filled in sterile cryotubes. Shipment was performed on dry ice. Here, the DNA was stored in the dark at a temperature below − 20 °C. The temperature was continuously monitored. The plasmids were provided in 1 $\times$ PBS buffer. The 1 $\times$ PBS buffer has a physiological pH of 7.4 and consists of Millipore water with NaCl (137 mmol/L), KCl (2.7  mmol/L), Na_2_HPO_4_ (10 mmol/L), KH_2_PO_4_ (1.8 mmol/L)^1^.

### Enzyme digests to generate open-circular and linear plasmids

To analyze the linearity of the signal intensities between supercoiled, open-circular and linear forms of plasmid DNA as measured by CGE, three types of samples were produced. Linear plasmids was produced by digest with *HindIII* (Jena Bioscience), while open-circular plasmids was produced *Nt.BspQI* (NEB). The third, untreated plasmid sample with a high degree of supercoiled plasmid DNA was treated equally to the both enzyme-digested samples. Therefore the same dilution and heating steps were performed during both treatments, but in this case just without any restriction enzyme present.

In detail: samples with each 50 μL of the vortexted, stock plasmid DNA solutions were diluted in 380 μL of ultrapure water (Merck Lichrosolv) and 50 μL of the reaction buffer were added (from NEB and Jena Bioscience respectively). The samples were vortexed again. Concentrations were checked by UV–Vis spectroscopy and it was controlled that the UV absorbance was the same before any enzyme was added. The UV–Vis measurements were performed with a Hamamatsu UV-2101 spectrometer and data was analysed with the the standard value of an absorbance at a wavelength of 260 nm of dsDNA = 1 at concentrations of 50 μg/mL. As reference the respective reaction-buffer water mixture was used. Afterwards, either 20 μL of *Nt.BspQI*, *HindIII* or water were added to the different samples. They were heated for either 60 min at 50 °C (*Nt.BspQI*) or 15 min at 37 °C (*HindIII*) and deactivated for 20 min at 80 °C, each.

**Fig. 8:**
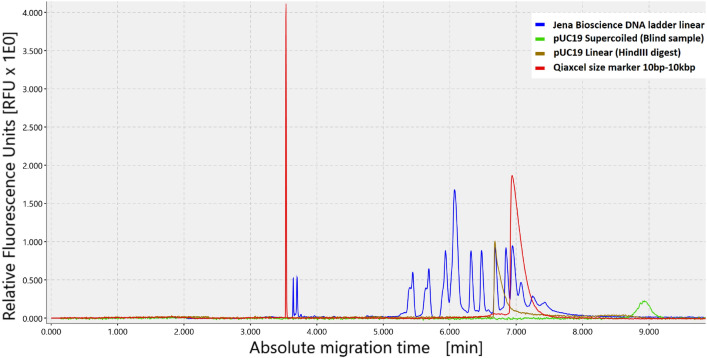
Plasmind pUC19 peak positions in CGE compared to a DNA ladder. Shown are supercoild pUC19 plasmid DNA (green), linearized pUC19 (brown), Qiaxcel 10 bp–10 kbp size marker (red) and Jena Bioscience linear *High range DNA ladder (blue)*. The maximum of the supercoilded DNA can be found at around 530 s and of the linearized DNA at around 400 s. Note that both plasmids DNA samples contain 2686 bp and that they differ only in their form. The linear form comigrates with the 3000 bp marker of the DNA ladder.

In case of the time dependent plasmid digest with *HindIII*, the experiments were performed with a stock solution prepared from 50 μL of the vortexted, stock plasmid DNA solutions were diluted in 380 μL of ultrapure water (Merck Lichrosolv) and 50 μL of Jena Bioscience reaction buffer. Ten aliquots of 40 μL were prepared and incubated simultaneously at 37 °C for 18 min. During this incubation 1 μL of *HindIII* was added to all samples (except to the blind sample) subsequently, at varying timesteps resulting in incubation times in presence of the enzyme between 0 and 18 min, as indicated in the legend of Fig. 7. After the incubation no deactivation was performed. The samples were centrifuged briefly for 10 s and measured directly by CGE after the digest. Following the first run a second run was directly performed afterwards to check for further digestion at room temperature. The Fluorescence High Range DNA ladder (linear, 50–10 kbp, 130 ng/μL stock concentration) from Jena Bioscience was used for assignment of the linear band (Fig. 8).

### Capillary gel electrophoresis

The capillary gel electrophoresis measurements were performed on a QIaxcel Advanced system (Qiagen-Hilden, Germany) in combination with the standard QIaxcel DNA Screening Kit^33^. The capillaries of this screening kit have an outer diameter of 1.5 mm and a distance of 130 mm between capillary entrance and measurement window.

Before the measurements all samples were diluted to achieve their final concentrations in either the QIaxcel provided buffer or 1 $\times$ PBS, vortexed for 3 s at 10 Hz, and centrifuged in a micro centrifuge for at least 30 s before the run. Cartridges were prepared according to the manufacturers instructions as described in the *QIaxcel DNA Handbook* shipped with the device. For analysis of the samples a customized running profile with the following parameters was used: A sample injection voltage of 2 kV, and varying sample injection times of 40 s, 60 s, 80 s a separation voltage of 4 kV, a total separation time of at least 1320 s, as indicated for each dataset. This custom profile with extended runtime for the DNA screening kit was obtained on request from the Qiagen support. When applied, an Qiaxcel Alignment marker 15 bp–10 kbp was used. After the run, data was exported to a CSV from from the Qiagen Screen Gel software and analysed by first performing either a background substraction by using the signal of the pure buffer, or when not needed, a linear background only. Afterwards, the regions assigned to the three different isoforms were integrated as marked by the three different colors in Fig. 1 This analysis was performed by a python script, reading the CSV files in a Pandas dataframe, substracting a linear background or the signal from the lane containing the buffer and afterwards integrating the region of the respective plasmid conformations. For the plasmid DNA pUC19 the regions for integration of the linear, supercoiled and open-circular form were centered around 400 s, 500 s and 750 s respectively. The exact position can differ somewhat due to varaition in migration speed between different capillaries, injection times and dilution buffer used. For the quantitative analysis only samples with an adequate SNR ratio and overall high signal intensity were included. If not indicated otherwise, the integrated areas were normalized on the sum obtained for all three conformations within each measurement. Uncertainties are given as standard deviation from the replicates of the same sample, measured with the same conditions.

Linear regression was performed with the *Python3 scipy.stats* module.

### Agarose gel electrophoresis

Agarose gel electrophoresis (AGE) was performed with a integrated Cyfox device (Sysmex, Germany). The DNA samples were vortexed for at least 3 s at 10Hz before the pipetting them into the respective lane of the gel. The measurements were performed with 1% Agarose gels (NEB precast gels) containing 1 × TAE buffer at 60 V corresponding to a field strength of 5 V/cm. The duration of each run was 30–60 min. For each gel 8 channels were loaded with 5 μL of DNA at 50 ng/μL in 5 μL of running buffer. The running buffer (1 $\times$ TAE) contained of 50 $\times$ SyBr Gold (Thermo) and 40 wt% sucrose (Sigma). The gels were imaged directly after the run within the Cyfox device. The AGE data was analysed by established procedures via multipeak fitting of gaussians to the different plasmid isoforms as described in previous work in detail^11,34^. If not indicated otherwise, all results were normalized on the sum of the peaks assigned to the different conformations. The DNA conformations were quantified by measuring the fluorescence intensity of the different bands assigned to the respective conformations. The given results were obtained by averaging over at least six samples for each datapoint. The attachment efficiency^35^ of SYBR Gold to the SC/CCC form of pUC19 compared to the open circular form is 1.05^34^, therefore no correction of the intensities values for the different plasmid forms is necessary.

## Data Availability

The data that support the findings of this study are available from the corresponding author upon request.
